# BolA family member 2 enhances cell proliferation and predicts a poor prognosis in hepatocellular carcinoma with tumor hemorrhage

**DOI:** 10.7150/jca.31829

**Published:** 2019-07-10

**Authors:** Jia Luo, Dong Wang, Sai Zhang, Kuan Hu, Haijun Wu, Juanni Li, Zhiming Wang, Yiming Tao

**Affiliations:** 1 Department of General Surgery, Xiangya Hospital, Central South University, Changsha 410008, China.; 2 Department of Surgery, Hunan Provincial Tumor Hospital, Changsha 410006, Hunan, China.; 3 Institute of Medical Sciences, Xiangya Hospital, Central South University, Changsha 410008, China.; 4 Department of Oncology, Xiangya Hospital, Central South University, Changsha 410008, China.; 5 Department of Pathology, Xiangya Hospital, Central South University, Changsha 410008, China.

**Keywords:** Hepatocellular carcinoma, BolA family member 2, Iron metabolism, Tumor hemorrhage, Tumor microenvironment

## Abstract

**Objective**: BolA family member 2 (BOLA2) is a novel gene highly associated with human hepatocellular carcinoma (HCC) progression. Tumor hemorrhage (TH) acts as a poor marker for HCC patients and is a community affair in the tumor microenvironment. In the present study, we examined a possible association between BOLA2 levels and HCC patients with TH.

**Methods**: The mRNA and protein levels of BOLA2 were determined in two independent cohorts of HCC specimens by quantitative real-time polymerase chain reaction (qRT-PCR) and immunohistochemistry (IHC) analysis, respectively. Survival curves and Cox regression models were used to evaluate the prognosis of HCC patients. The CRISPR/Cas9 system was used to knock out BOLA2 in HCC cells, and the functional role of BOLA2 in HCC cell proliferation *in vitro* and growth *in vivo* was examined.

**Results**: BOLA2 mRNA expression is significantly higher in HCC tumour tissue than in nontumour tissue. Immunohistochemistry analysis of HCC tissues showed that BOLA2 protein was significantly correlated with TH, a more metastatic phenotype and worse HCC survival. The potential clinical relevance of BOLA2 expression and TH was validated by a Cox regression model. Furthermore, loss-of-function studies determined that BOLA2 plays critical roles in promoting iron overload, tumor growth and TH. Bioinformatics analysis from Gene Expression Profiling Interactive Analysis (GEPIA) revealed that BOLA2 is closely associated with the activation of p62-Keap1 signalling and ATG4B expression. These results were confirmed by immunohistochemistry analysis in HCC tissues.

**Conclusions**: Our results suggest that BOLA2 plays an important role in cancer biology and is an independent predictor of prognosis in HCC.

## Introduction

Hepatocellular carcinoma (HCC) is one of the most malignant tumors and has an extremely poor prognosis 1. The tumor microenvironment plays a critical role in promoting HCC progression and metastasis 2. As an underlying key indicator of a fast-growing tumor, tumor hemorrhage (TH) is associated with progression and metastasis 3. TH has been demonstrated to be a poor marker for HCC patients 4. Therefore, determining the underlying mechanistic basis of these pathogenetic changes in HCC could greatly benefit the efficacy of the clinical treatment of HCC.

BolA family member 2 (BOLA2) is a nonclassical secreted member of the BolA protein family that also contains BOLA1, BOLA2 and BOLA3 5. Previous studies have revealed that BOLA2 functions as a key regulatory for intracellular iron homeostasis in the microenvironment 6, 7. Increasing evidence has revealed that iron homeostasis dysregulation is linked to cancer initiation and development 8, 9. BOLA2 was also reported to potently promote c-Myc's oncogenic activity in liver cancer 10. Although its expression was reported to be elevated during tumorigenesis, BOLA2 has not been extensively studied in cancer, including HCC.

For the first time, we investigated the expression of BOLA2 expression in both HCC recurrence and metastasis and evaluated the significance of BOLA2 in the prediction of HCC prognosis, especially TH. We analysed the expression and functions of BOLA2 in HCC cell growth and TH. Our findings suggest that BOLA2 can be a potential therapeutic target for the treatment of HCC metastasis.

## Materials and Methods

### Patient populations and follow-up

For the use of clinical HCC tissues for research purposes, informed consent was obtained from each HCC patient before hepatic resection. All patients and the study protocol were approved by the Ethics Committee of Central South University (Xiangya Hospital, China). Two independent cohorts of HCC specimens were examined in this study. In the training cohort (n=96), fresh matched specimens of HCC and adjacent non-tumor tissue (NT) were randomly collected from HCC patients undergoing hepatic resection between August 2011 and October 2011 in the Department of Hepatobiliary Surgery, Xiangya Hospital, China. In the validation cohort (n=175), paraffin-embedded specimens were randomly collected from HCC patients undergoing curative resection in Xiangya Hospital from March 2012 to November 2012. HCC specimens accompanied with TH were reviewed by two independent pathologists in our hospital. The following procedures from our previous study were adopted 11. Follow-up of HCC patients was terminated on 31 January 2017. All research protocols strictly complied with REMARK guidelines for reporting prognostic biomarkers in cancer 12.

### HCC cell lines

HepG2, Hep3B, SNU449 and PLC/PRF5 cells were obtained from the American Type Culture Collection (ATCC, Rockville, MD). L02, SMMC7721, Bel7402 and Huh7 cells were purchased from the Cell Bank of Typical Culture Preservation Committee of Chinese Academy of Science (Shanghai, China). L02 is a normal liver cell line that is usually used as a control cell in cancer research. MHCC97L cell lines were provided by the Liver Cancer Institute of Zhongshan Hospital of Fudan University (Shanghai, China). The characteristics and proteome analysis of MHCC97L have been previously described 13. All cell lines were routinely authenticated by STR genotyping and mycoplasma detection. Cells were stored in liquid nitrogen and cultured in 5% CO_2_ at 37°C with high-glucose Dulbecco's modified Eagle media (GIBCO, Grand Island, NY) supplemented with penicillin (100 IU/mL), streptomycin (100 mg/mL) and 10% FBS (GIBCO).

### Quantitative RT-PCR in clinical HCC specimens

The procedures of SYBR green fluorescent-based quantitative reverse transcriptase polymerase chain reaction (qRT-PCR) were performed as previously described 11. Actin was used as a control for BOLA2 gene detection. The oligonucleotide sequences for BOLA2 and β-actin primers were as follows: BOLA2, 5'-CTGTAGCTTCCGAGTCCTG, 3'-TTCAAAGGCATGGATGTGC; and β-actin, 5'-GGACTTCGAGCAAGAGATGG, 3'-AGCACTGTGTTGGCGTACAG.

### Immunohistochemistry (IHC) analysis

IHC analysis and scoring were performed as previously described 14. A BOLA2 polyclonal antibody (GeneTex, MA, USA, Cat. # GTX51984) diluted at 1:1000 was used as the primary antibody. A peroxidase-labelled polymer was conjugated to goat anti-rabbit IgG (DAKO Corporation, Carpinteria, CA, Cat. # K5007) for 30 minutes at room temperature, and color was developed using 3, 3'-diaminobenzidine (DAB) solution. BOLA2 expression was assessed when cytoplasmic or membranous staining was observed.

### Western blot analysis

Western blot analysis were performed as previously described 15. The antibody dilutions were 1:1,000 for the BOLA2 polyclonal antibody (Cat. # ab169481, Abcam), 1:1,000 for the p62 polyclonal antibody (ABGNT, Cat. # AP2183B), 1:1,000 for the Keap1 polyclonal antibody (ABGNT, Cat. # ALS15665), and 1:5,000 for the β-actin mouse monoclonal antibody (Sigma-Aldrich, Cat. # A1978). All Western blot images were analysed with ImageJ software (NIH) to determine signal intensity.

### Establishment of BOLA2-knockout HCC cell lines

The characteristics of HCC Hep3B cells with high intracellular iron content have been described in the literature 16. BOLA2-deficient Hep3B cells were established using the CRISPR/Cas9 system. The gRNA was selected with the assistance of the CRISPR design tool according to a standard protocol 17. The sgRNA sequences of BOLA2 were 5'-CATGGCAAGCGCGAAAAGCC-3'; and bottom strand: 5'-TCACCACATGCTCCGCCTCC-3'. For control cells, no guide RNA was inserted. Each experimental replicate was performed using the HCC cell line Hep3B.

### Cell proliferation assay

Eight hundred Hep3B cells per well were grown in 96-well plates, and a cell proliferation assay was performed using a Cell Counting Kit-8 (Med Chem Express, USA, Cat. # HY-K0301) according to the manufacturer's protocol. Each experiment was repeated three times.

### Colony-forming assay

Hep3B cells were seeded into six-well plates (1000 cells/well). After incubation for 14 days, the colonies that formed were washed and then stained with 0.2% crystal violet (Fisher Scientific, Asheville, NC, Cat. # R40053). The colonies were quantified from three independent experiments per treatment group.

### Tumor xenograft model

All animal work was conducted in accordance with protocols approved by the Animal Care and Use Committee at Central South University, China. A subcutaneous HCC mouse model was established according to an existing protocol 18. BOLA2-knockout Hep3B cells (2×10^6^ cells/mouse) were injected subcutaneously into the right axilla (200 μl). All nude mice were sacrificed six weeks after tumor implantation. Prussian blue staining (Sigma-Aldrich, Cat. # 03899) was performed on sections of each transplanted tumor lesion. In addition, IHC staining against BOLA2, p62 and Ki-67 was performed on paraffin sections.

### Bioinformatics prediction

We used the Oncomine database (www.oncomine.com) to predict the levels of BOLA2 mRNA in HCC and normal tissues. Then, Gene Expression Profiling Interactive Analysis (GEPIA) (http://gepia.cancer-pku.cn/) was employed to forecast a potential correlation between BOLA2 mRNA expression levels and the overall survival of HCC patients 19. Images of BOLA2 protein expression in HCC specimens and normal liver tissues were obtained from the Human Protein Atlas (www.proteinatlas.org) online database 20. Tumor Immune Estimation Resource (TIMER) (https://cistrome.shinyapps.io/timer/) was used to study the correlation between the BOLA2 gene and a pair of user-defined genes in liver hepatocellular carcinoma (LIHC) 21. A protein-protein interaction network of BOLA2 highly correlated genes was generated by the STRING database (http://string-db.org).

### Statistical analysis

Statistical analyses were performed using SPSS version 20.0 (SPSS, Chicago, IL, USA) and GraphPad Prism software (GraphPad Software, San Diego, CA). Quantitative values are presented as the mean ± SD or median (range). Paired *t* tests and Student's tests were used for paired and unpaired continuous data, respectively. The *χ^2^* test was applied for categorical data. The cumulative overall survival (OS) and recurrence-free survival (RFS) were evaluated using the Kaplan-Meier method and the log-rank test. A Cox proportional hazards regression model was used to determine whether BOLA2 expression is an independent prognostic indicator. Statistically significant changes are indicated with asterisks. *= (*P* < 0.05); **= (*P* < 0.01); *** = (*P* <0.001).

## Results

### Overexpression of BOLA2 correlated with poor survival in HCC

**Fig 1:**
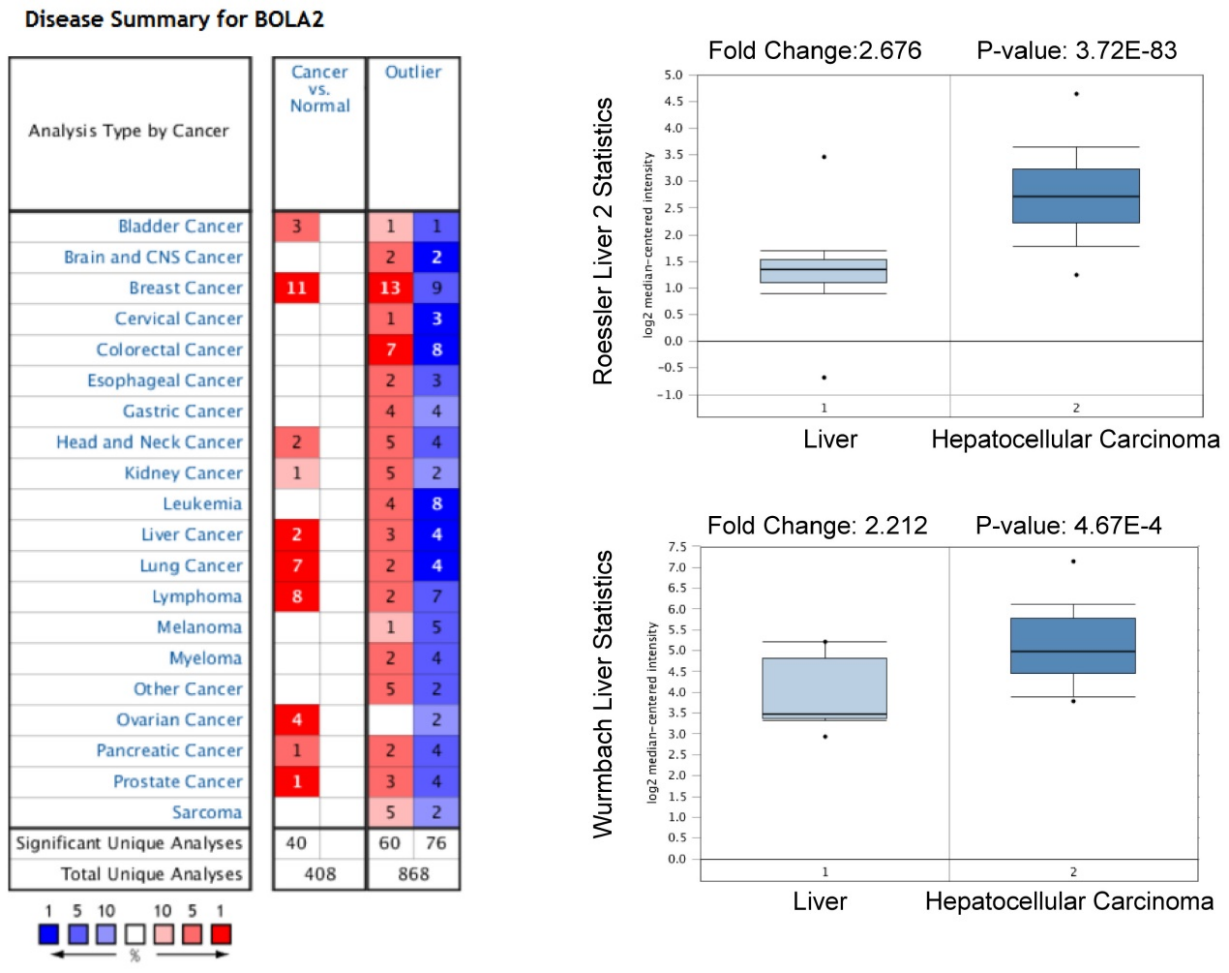
High expression levels of BOLA2 mRNA in HCC predicted by the Oncomine database. BOLA2 mRNA levels in Roessler Liver2 (GEO: GSE 14520/GPL3921) and Wurmbach Liver (GEO: GSE 6764) grouped by HCC and normal liver in the Oncomine database.

**Fig 2:**
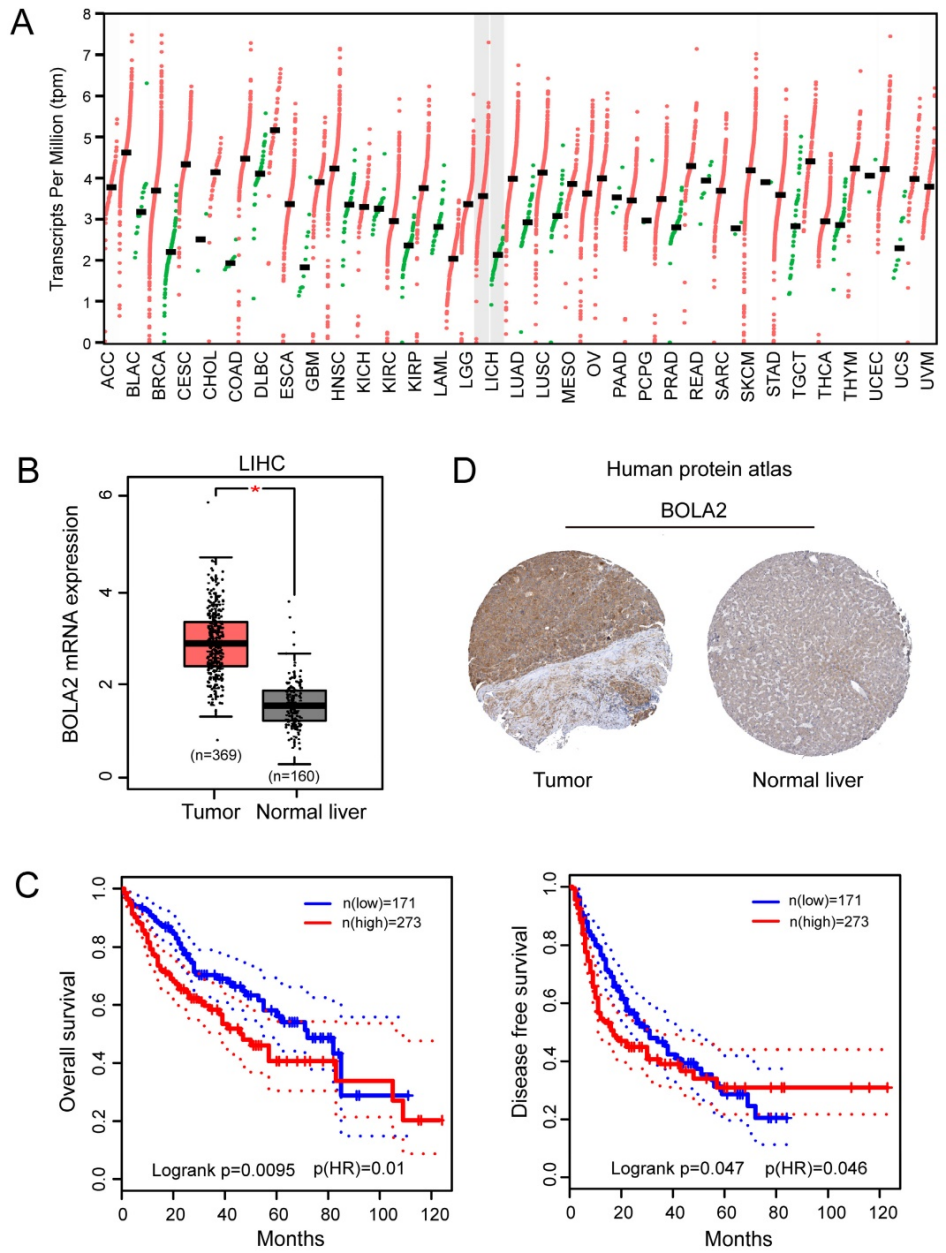
BOLA2 expression level is significantly increased in HCC and is associated with poor clinical outcomes. (A) Differential expression of BOLA2 between tumor and adjacent normal tissues from various types of cancer. (B) BOLA2 gene expression was significantly increased in HCC (n=369) compared with corresponding normal liver tissues (n=160). Data were extracted from the GEPIA web server. (C) Kaplan-Meier curves stratified by BOLA2 mRNA expression. Overall survival and disease-free survival data were generated from the GEPIA web server. (D) BOLA2 protein expression in normal liver tissues and HCC specimens. Images were obtained from the Human Protein Atlas online database.

We performed a pan-cancer analysis of the expression of BOLA2 genes in normal and cancerous tissues by using the Oncomine database and found that BOLA2 was increased in most types of solid tumors, including breast, colorectal, pancreatic, and liver (Figure 1). Similar results were found through the online GEPIA database (Figure 2A). BOLA2 mRNA was significantly increased in HCC compared with normal liver tissues (*P*<0.05, Figure 2B). High levels of BOLA2 mRNA also strongly correlated with a higher HCC stage, worse survival (*P* = 0.0095) and disease-free survival (*P* = 0.047) (Figure 2C). In addition, the human protein atlas verified a similar expression trend of BOLA2 protein within HCC samples (Figure 2D).

### Association between BOLA2 and TH

**Fig 3:**
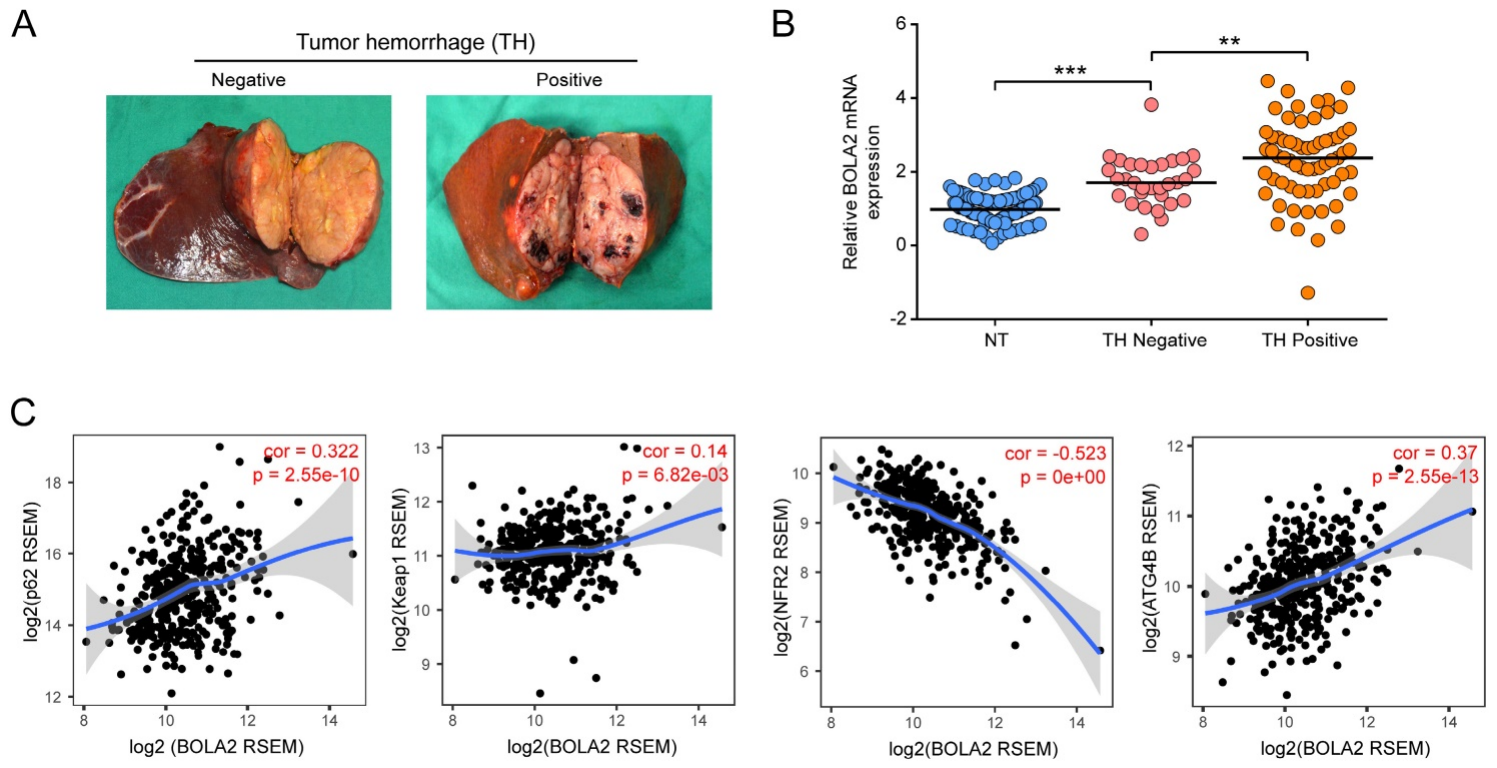
Increased expression of BOLA2 mRNA is related to a high risk of TH. (A) Representative gross specimens of HCC with/without TH. (B) BOLA2 expression in a NT, a TH-negative tumor, and a TH-positive tumor. ** P < 0.01. *** P <0.001. Abbreviations: NT, nontumor tissues. (C) Spearman's correlation analysis of tumor oxidative stress markers, including p62, Keap1, Nrf2, and ATG4B, with BOLA2 expression in the HCC profiles of the TCGA dataset.

We examined the relative transcription levels of BOLA2 by qRT-PCR in tumor tissues and corresponding nontumour tissue (NT) in a cohort of 96 HCC patients. The mRNA expression of BOLA2 in the HCC tumor tissues of the training cohort significantly correlated with the clinicopathological features of tumor invasion and aggression, such as venous invasion, satellite nodules, tumor hemorrhage (TH), tumor differentiation and HCC stage (Table 1). Intriguingly, when 96 HCC tumors were classified into subgroups by TH or not (Figure 3A), HCC tumors with TH showed even higher expression of BOLA2 (1.748 ± 0.118 versus 2.317 ± 0.135, *P*<0.01; Figure 3B). TH indicated a worse tumor immunological microenvironment 4, 22. As shown in Figure 3C, by using TIMER, a positive correlation was observed between BOLA2 mRNA expression and defined reactive oxygen species (ROS) modulator genes in HCC, such as p62, autophagy-related 4B (ATG4B) and Kelch-like ECH-associated protein 1 (Keap1), whereas BOLA2 mRNA expression was inversely correlated with nuclear factor erythroid 2-related factor 2 (NRF2). These results suggest that BOLA2 expression correlates with oxidative stress and TH.

### BOLA2 expression predicts clinical outcome in HCC

**Fig 4:**
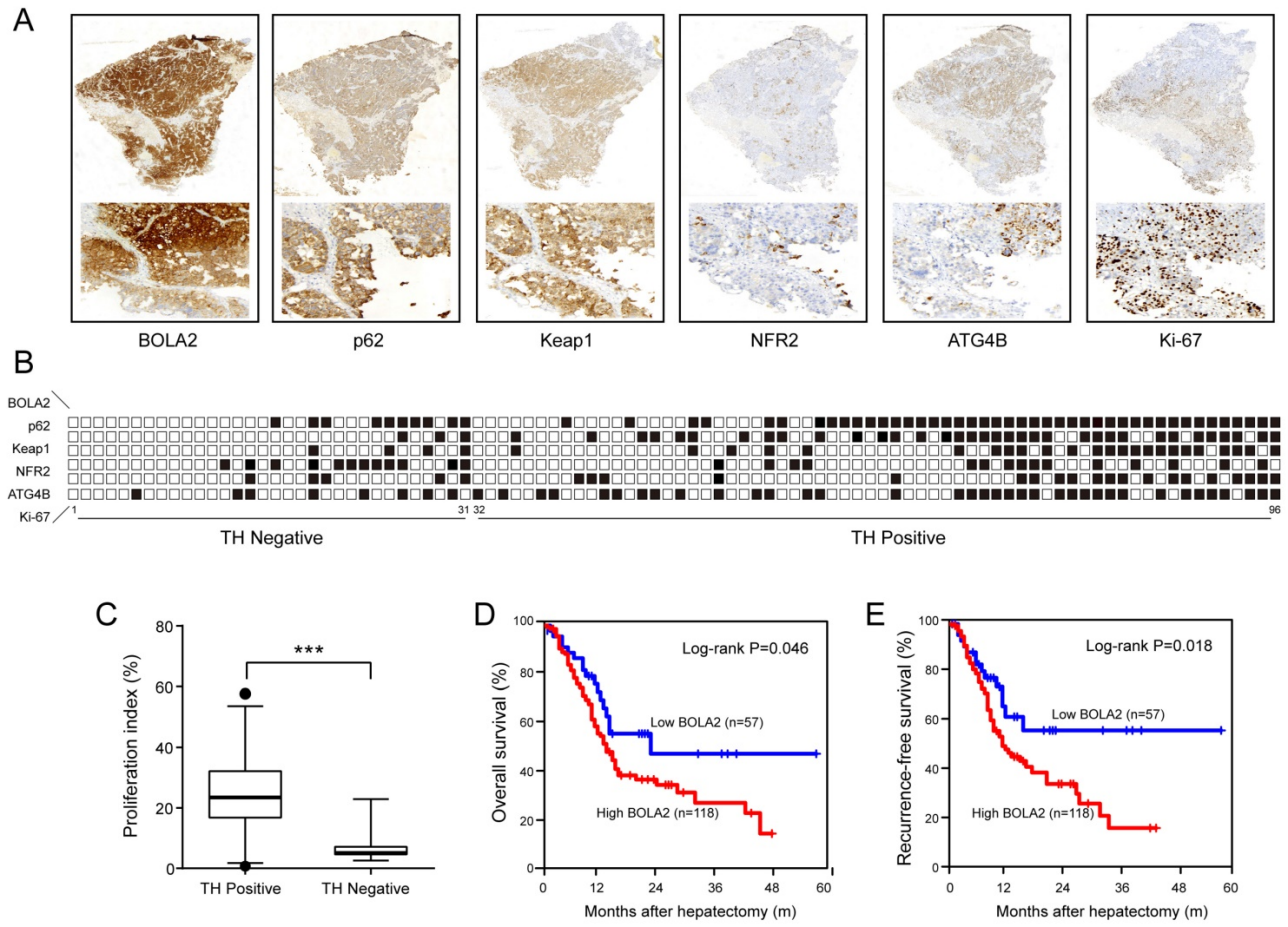
(A) A summary of the immunohistochemical staining results of the training cohort. Membrane and cytoplasmic expression of BOLA2, p62, and Keap1 and the nuclear expression of NRF2 and Ki-67 were observed. (B) The samples are sorted from left to right in ascending order based on the presence of TH. The positive samples are indicated by black boxes. HCC patients with TH and lower BOLA2 expression had higher levels of NRF2, whereas patients with higher BOLA2 expression had increased ATG4B, p62 and Keap1 levels. (C) Quantification of Ki-67 in HCC tissues with or without TN. Ki-67-positive cells are numbered, and box plots show the median, 25th and 75th percentiles, and minimum and maximum values. (D and E) Kaplan-Meier curves depicting overall survival (OS) and recurrence-free survival (RFS) according to the expression levels of BOLA2 in the validation cohort (n=175).

To confirm the above results, in a training cohort of HCC samples, we validated the expression of BOLA2, p62, Keap1, NRF2, and ATG4B and the proliferation marker (Ki-67) using immunohistochemistry (Figure 4A). HCC patients with TH and lower BOLA2 expression had higher levels of NRF2, whereas patients with higher BOLA2 expression had increased ATG4B, p62 and Keap1 levels (Figure 4B). Furthermore, HCC patients with TH showed even higher Ki-67 expression (Figure 4C). Moreover, the expression levels of BOLA2 protein were confirmed in another validation cohort containing 175 HCC patients (Table 2). Cox's multivariate analysis indicated that BOLA2 expression was an independent positive prognostic factor in the training cohort (HR=2.108, 95% CI 1.541 to 6.067; Table 3). The BOLA2^high^ expression group exhibited worse overall survival (OS) and shorter recurrence-free survival (RFS) than the BOLA2^low^ expression group (Figure 4D and 4E). The 1-, 3-, and 5-year OS rates in the high expression group were significantly lower than those in the low expression group (60.2% versus 79.8%, 28.4% versus 48.5%, and 18.2% versus 45.7%, respectively). The 1-, 3-, and 5-year RFS rates were markedly lower in the high expression group than in the low expression group (52.2% versus 75.6%, 18.4% versus 39.7%, and 16.6% versus 43.4%, respectively). Taken together, these results suggest that the value of BOLA2 may be a clinical predictor of HCC prognosis.

### BOLA2 knockout suppresses HCC cell proliferation, tumor growth and TH

**Fig 5:**
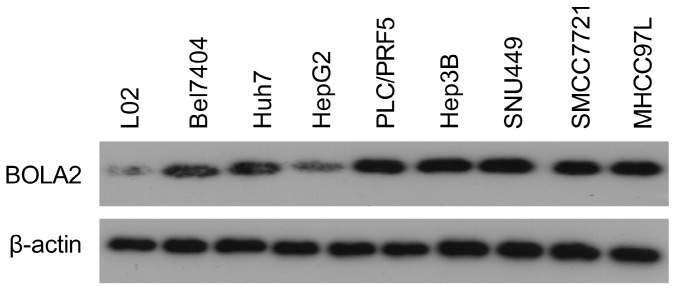
Western blot analysis was used to examine the expression of BOLA2 in HCC cell lines versus immortalized human hepatocytes (L02).

**Fig 6:**
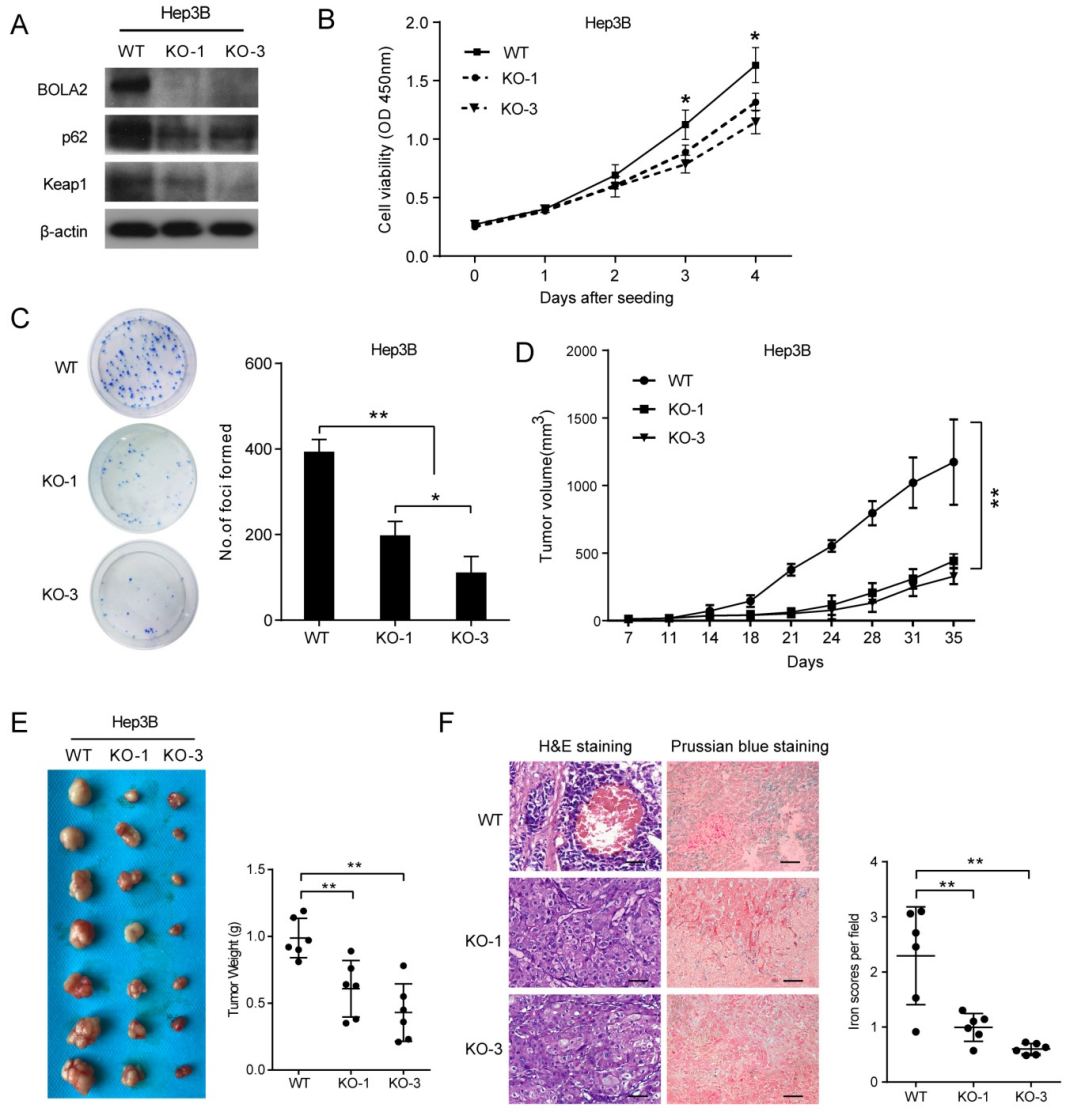
BOLA2 knockout reduces the tumorigenicity of HCC cells *in vitro* and in a xenograft model. (A) BOLA2 knockout by CRISPR/Cas9 technology in Hep3B cells was confirmed by Western blot analysis. The activities of p62 and Keap1 were reduced in BOLA2-deficient Hep3B cells. (B) BOLA2 knockout slows cell proliferation. (C) Colony formation assays were performed in the three BOLA2-deficient colonies derived from Hep3B and WT cells. The values are expressed as the mean ± SD of three independent experiments. (D) Tumor growth curves in the three groups are shown on the indicated days after Hep3B cell (WT, KO-1, and KO-3) injection. The xenograft tumor volumes of each group were measured two times a week. (E) Representative images of subcutaneous tumors in nude mice injected with the indicated cells. Final tumor weights are summarized in a dot chart. The average tumor weight is expressed as the mean ± SD of 6 mice. (F) H&E staining demonstrated that the KO of BOLA2 inhibited the TH phenotype of HCC *in vivo* (left panel). Corresponding Prussian blue staining shows iron particles scattered in the central part of the tumor with TH. The iron scores of the xenograft tumors were calculated and are depicted in the bar chart (right panel).

The protein levels of BOLA2 among eight established HCC cell lines were compared with the immortalized human hepatocyte cell line L02. BOLA2 expression was higher in HCC cells than in L02 cells (Figure 5). We knocked out BOLA2 in Hep3B cell lines using CRISPR/Cas9 technology and evaluated the effects of BOLA2 in these cells. BOLA2 modulation exhibited significant effect on the protein expression of p62 and Keap1 in BOLA2-deficient Hep3B cells (Figure 6A). Cell proliferation assays revealed that BOLA2 knockout potently slowed cell proliferation compared with the WT cell group (Figure 6B). Compared with WT cells at day 4, the suppressive rates were 37.5% (KO-1) and 33.3% (KO-3) in the three BOLA2-deficient colonies derived from Hep3B cells. Cell colony assays further confirmed the similar suppressive effects of BOLA2 knockout (Figure 6C), with inhibitory rates of 45.3% and 33.6% in Hep3B KO-1 and KO-3 cells, respectively. These findings indicate the oncogenic role of BOLA2 in HCC tumor progression.

Next, we attempted to determine whether BOLA2 knockout had the same suppressive effects on HCC tumor growth *in vivo*. Subcutaneous xenograft animal studies were performed to further investigate the tumorigenicity of BOLA2* in vivo*. Compared to the WT group, the knockout of BOLA2 in HCC cells induced strong tumor growth suppression (Figure 6D). The average tumor weights in the KO-1, KO-3, and WT groups were 0.61±0.08 g, 0.43±0.08 g, and 0.98±0.06 g, respectively (Figure 6E). We found that xenografts derived from Hep3B-KO-BOLA2 cells exhibited decreased TH (Figure 6F, left panel). Intracellular iron is essential for HCC cell growth 23. Another intriguing finding was that after the injection of Hep3B-KO-BOLA2 cells, the iron scores decreased markedly in the transplanted tumors of nude mice according to the histological examination of Prussian blue staining (0.61±0.04 vs. 0.99±0.10 vs. 2.29±0.36, Figure 3F, right panel). This observation led us to further explore the mechanism by which BOLA2 regulates the growth of HCC (Figure 6F, right panel).

### Involvement of p62-Keap1 signalling in BOLA2-mediated HCC cell proliferation

**Fig 7:**
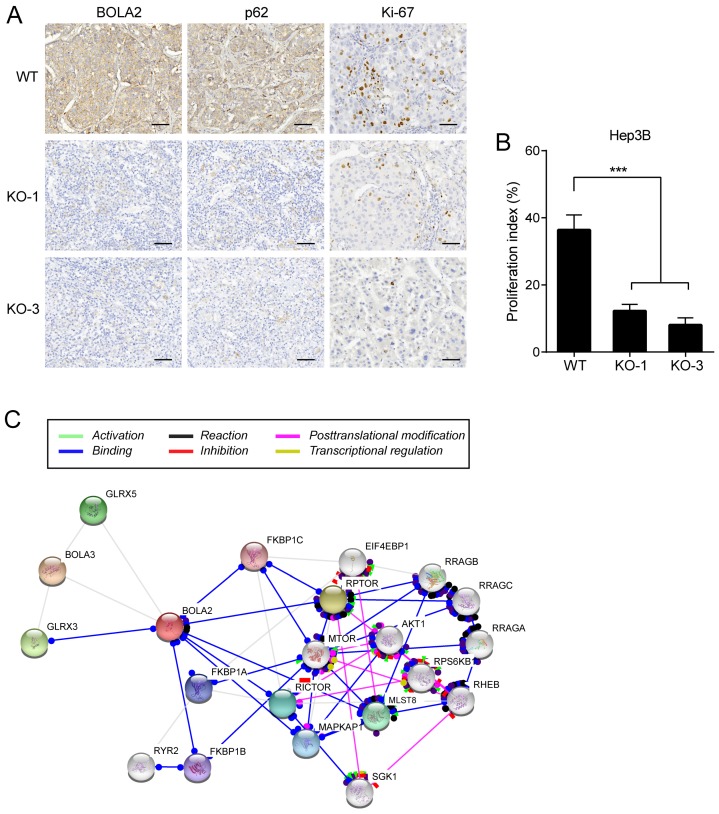
(A) Representative immunohistochemistry staining of BOLA2, p62 and Ki-67 in transplanted tumor sections from Hep3B cells (WT, KO-1, and KO-3) injected into nude mice (×400 magnification). Scale bar: 100 μm. (B) Comparison of Ki-67 staining in the transplanted tumors. (C) STRING database analysis of the PPI network for BOLA2. Interactions between 20 hub genes are illustrated with the cut-off criterion of a combined score =0.7. Network nodes represent proteins and edges represent protein-protein associations.

IHC staining was performed on serial sections of Hep3B cell transplantation tumors, and the protein expression of BOLA2 and p62 was significantly lower in the Hep3B-KO-BOLA2 group than in the WT group (Figure 7A). Proliferating cells were quantified by counting the number of Ki-67-positive cells; more Ki-67-positive cells were observed in WT tumors than in Hep3B-KO-BOLA2 tumors (Figure 7B). STRING was used to reveal the interactions of the BOLA2 pathway in HCC (Figure 7C), and a subnetwork of 20 genes was presented that included mTOR, AKT1, and MAPKP1, which are implicated in regulating tumor growth 24, 25. High levels of p62 expression are needed for the activation of NRF2 or mTORC1, the induction of c-Myc, and the protection of HCC-initiating cells from oxidative stress-induced cell death 26. Thus, we speculate that BOLA2 might promote the development of HCC and maintain cancer cell growth under metabolic stress conditions.

## Discussion

HCC is highly lethal because of its aggressive metastasis and the minimally effective strategies available against metastasis 27. HCC is characterized by its fast growth and multiple factors, including genetic and epigenetic alterations that cause uncontrolled cellular proliferation and metastasis. In the current study, BOLA2 overexpression was positively correlated with venous invasion, TH, satellite nodules, and TNM stage. Moreover, the survival analysis showed that HCC patients with BOLA2^high^ expression had shorter OS than those with BOLA2^low^ expression. We hypothesized that BOLA2 might act as a tumor promoter to enhance HCC development. To confirm this hypothesis, functional studies showed that the knockout of BOLA2 reduced HCC cell proliferation *in vitro* and decreased tumor growth subsequent to TH* in vivo*. Similar to other investigations that reported the rapid growth rate in HCC, BOLA2 overexpression enhanced proliferation and colony formation in HCC cells, highlighting its importance in HCC progression.

We previously demonstrated that TH was involved in fast-growth HCC. The precise mechanism underlying this BOLA2-mediated TH of tumor growth remains to be determined. BOLA2 expression is regulated by at least four mechanisms: (i) specific in-frame fusion transcript regulation, which controls the copy number; (ii) monothiol CGFS glutaredoxin binding partners 28; (iii) a glutaredoxin-3 (GRX3)-dependent anamorsin maturation pathway 29; and (iv) as a c-Myc-targeted gene in HCC 10. In this work, we performed a TIMER analysis, and the results indicated that BOLA2 is highly associated with p62 expression in the tumor immunological microenvironment. A recent study showed that activation of the p62-Keap1-NRF2 pathway protects against ferroptosis in HCC cells 30. The Keap1-Nrf2 system and autophagy are both involved in the oxidative stress response, metabolic pathways, and innate immunity, and the dysregulation of these processes is associated with pathogenic processes 31. Therefore, we speculate that BOLA2 contributes to uncontrolled cellular proliferation, which may cause HCC development.

It is widely accepted that carcinogenesis is a multistep process that involves cell proliferation, adhesion, growth, and metabolism 32. Increasing evidence has revealed alterations in iron metabolism in HCC that facilitate cancer cell growth 33. Recent evidence suggests that the iron-mediated production of reactive oxygen species promoted autophagy in cancer stem cells 34. TH is a very complex process in the tumor microenvironment and involves hypoxia, cell death, necrosis, the immune response, angiogenesis and nutrient-generating autophagy, etc. 3. We found the overexpression of BOLA2 in human HCC tumors with TH; these findings reveal BOLA2 as a potential target against HCC.

Iron is essential for the proliferation of normal and neoplastic cells. Many cancers exhibit an increased requirement for iron, because the need for iron as a regulator is essential for growth and proliferation 9. Excess liver iron (iron overload) is one of the major risks leading to increased HCV hepatitis, fibrosis, cirrhosis, and HCC 35. Recent studies have reignited interest in the deregulation of iron homeostasis as an anticancer therapy 36, 37. Further studies of BOLA2 in HCCs will be of interest to clarify its precise role in tumor growth and TH.

## Conclusions

Collectively, the present results indicate that BOLA2 is highly expressed in tumor tissues and is significantly correlated with a poor prognosis in HCC patients. Moreover, BOLA2 serves as a key emerging molecule in HCC cell growth and TH. These findings have important implications for identifying new therapeutic targets in HCC.

## Figures and Tables

**Table 1:** Correlation between BOLA2 mRNA expression and clinicopathological characteristics in 96 HCC patients

Clinical and pathological variable		BOLA2 mRNA		
		Low expression (n=39)	Overexpression (n=57)	*P* value†
Age, years	≤60	22	33	0.885
	>60	17	24	
Sex	Male	26	47	0.075
	Female	13	10	
Child-Pugh score	A	25	46	0.069
	B	14	11	
Albumin, g/L	≤35	7	4	0.099
	>35	32	53	
AFP, (ng/mL)	≤20	18	19	0.205
	>20	21	38	
Liver cirrhosis	Absent	7	5	0.182
	Present	32	52	
Tumor size, (cm)	≤5	17	16	0.116
	>5	22	41	
Tumor number	Single	25	26	0.075
	Multiple	14	31	
Tumor encapsulation	Complete	18	16	0.069
	None	21	41	
Satellite nodules	Absent	23	18	**0.008**
	Present	16	39	
Venous invasion	Absent	25	10	**<0.0001**
	Present	14	47	
Tumor differentiation	I-II	8	24	**0.028**
	III-IV	31	33	
Tumor hemorrhage	Absent	21	10	**0.0002**
	Present	18	47	
BCLC stage	A	23	13	**0.0003**
	B-C	16	44	
TNM stage	I	18	12	**0.009**
	II-III	21	45	

† Fisher's exact test or the chi-square test. A significant difference is shown in bold.

**Table 2:** Correlation between BOLA2 protein and clinicopathological characteristics in 175 HCC patients

Clinical and pathological variable		BOLA2 protein		
		Low expression (n=57)	High expression (n=118)	*P* value
Age, years	≤60	36	64	0.264
	>60	21	54	
Sex	Male	47	86	0.165
	Female	10	32	
Child-Pugh score	A	38	93	0.083
	B	19	25	
Albumin, g/L	≤35	9	11	0.208
	>35	48	107	
AFP, (ng/mL)	≤200	26	41	0.258
	>200	31	71	
Liver cirrhosis	Absent	10	12	0.168
	Present	47	106	
Tumor size, (cm)	≤5	24	36	0.130
	>5	33	82	
Tumor number	Single	36	57	0.065
	Multiple	21	61	
Tumor encapsulation	Complete	23	39	0.344
	None	34	79	
Satellite nodules	Absent	42	33	**<0.0001**
	Present	15	85	
Venous invasion	Absent	37	27	**<0.0001**
	Present	20	91	
Tumor differentiation	I-II	9	49	**0.001**
	III-IV	48	69	
Tumor hemorrhage	Absent	27	30	**0.004**
	Present	30	88	
BCLC stage	A	31	35	**0.002**
	B-C	26	83	
TNM stage	I	26	29	**0.005**
	II-III	31	89	

A significant difference is shown in bold.

**Table 3:** Univariate and multivariate analyses of RFS and OS prognostic factors in HCC patients (n=175).

	RFS		OS	
Clinicopathologic variable	HR (95% CI)	*P*	HR (95% CI)	*P*
**Univariate analysis**†				
Gender (male vs. female)	1.061 (0.867-1.584)	0.215	1.106 (0.923-1.316)	0.135
Age, years (> 60 vs. ≤ 60)	1.215 (0.986-1.764)	0.085	1.161 (0.952-1.417)	0.141
HBsAg (positive vs. negative)	1.298 (0.981-1.992)	0.064	1.135 (0.869-1.538)	0.061
Albumin, g/L (≤35 vs. >35)	1.052 (0.897-1.963)	0.089	1.195 (0.995-1.436)	0.056
Child-Pugh classification (B vs. A)	1.231 (1.053-1.768)	**0.037**	1.292 (1.084-1.852)	**0.043**
Liver cirrhosis (presence vs. absence)	1.312 (1.097-1.635)	**0.016**	1.369 (1.106-3.145)	**0.013**
Serum AFP level, ng/mL (> 20 vs. ≤ 20)	1.452 (1.079-1.894)	**0.036**	1.209 (1.012-2.174)	**0.046**
Tumor diameter, cm (> 5 vs. ≤ 5)	1.554 (1.116-3.425)	**0.012**	1.405 (1.106-2.642)	**0.021**
Tumor number (multiple vs. single)	1.659 (1.107-2.827)	**0.008**	1.554 (1.106-2.993)	**0.014**
Tumor encapsulation (none vs. complete)	1.152 (0.932-1.436)	0.072	1.304 (1.046-2.214)	**0.025**
Vascular invasion (presence vs. absence)	2.113 (1.462-4.834)	**<0.0001**	2.056 (1.279-6.164)	<0.0001
Tumor differentiation (III/IV vs. I/II)	1.478 (1.187-1.769)	**0.022**	1.352 (0.994-1.967)	0.069
Satellite nodules (presence vs. absence)	1.841 (1.267-3.156)	**0.004**	1.731 (1.036-1.948)	**0.010**
TH (presence vs. absence)	1.947 (1.296-6.324)	**0.003**	1.874 (1.236-3.524)	**0.007**
TNM stage (II/III vs. I)	1.212 (1.067-1.836**)**	**0.040**	1.546 (1.142-2.284)	**0.018**
BOLA2 expression level (high vs. low)	2.024 (1.186-3.895)	**0.002**	1.942 (1.246-4.537)	**0.005**
**Multivariate analysis**†				
Gender (male vs. female)	NA		NA	
Age, years (> 60 vs. ≤ 60)	NA		NA	
HBsAg (positive vs. negative)	NA		NA	
Albumin, g/L (≤35 vs. >35)	NA		NA	
Child-Pugh classification (B vs. A)	1.078 (0.967-1.842)	NS	1.208 (0.997-1.463)	NS
Liver cirrhosis (presence vs. absence)	1.102 (0.954-1.863)	NS	1.398 (0.981-1.992)	NS
Serum AFP level, ng/mL (> 20 vs. ≤ 20)	1.135 (0.869-1.538)	NS	1.364 (0.967-1.842)	NS
Tumor diameter, cm (> 5 vs. ≤ 5)	1.195 (0.995-1.436)	NS	1.154 (0.859-1.729)	NS
Tumor number (multiple vs. single)	1.874 (1.236-3.524)	**0.007**	1.768 (1.023-3.969)	**0.019**
Tumor encapsulation (none vs. complete)	NA		1.075 (0.951-1.375)	NS
Venous invasion (presence vs. absence)	2.152 (1.336-5.231)	**<0.0001**	1.994 (1.253-4.564)	**0.002**
Tumor differentiation (III/IV vs. I/II)	1.208 (0.946-1.764)	NS	NA	
Satellite nodules (presence vs. absence)	1.679 (1.214-3.216)	**0.021**	1.616 (1.207-3.542)	**0.024**
TH (presence vs. absence)	2.342 (1.678-7.429)	**<0.0001**	1.856 (1.246-5.682)	**0.005**
TNM stage (II/III vs. I)	1.229 (1.016-1.488)	**0.035**	1.231 (1.084-1.796)	**0.041**
BOLA2 expression level (high vs. low)	2.108 (1.541-6.067)	**0.004**	2.253 (1.341-6.568)	**<0.001**

Abbreviations: HCC, hepatocellular carcinoma; RFS, recurrence-free survival; OS, overall survival; HR, hazard ratio; CI, confidential interval; NA, not adopted; HBsAg, hepatitis B surface antigen; AFP, α-fetoprotein; TH, tumor hemorrhage; TNM, tumor-node-metastasis, unless otherwise indicated. A significant difference is shown in bold. † Cox proportional hazards regression.

## Abbreviations

HCC: hepatocellular carcinoma
BOLA2: BolA family member 2
TH: tumor hemorrhage
qRT-PCR: quantitative real-time polymerase chain reaction
IHC: immunohistochemistry
GEPIA: Gene Expression Profiling Interactive Analysis
TIMER: Tumor Immune Estimation Resource
OS: overall survival
RFS: recurrence-free survival
